# Insulin-induced genes INSIG1 and INSIG2 mediate oxysterol-dependent activation of the PERK–eIF2α–ATF4 axis

**DOI:** 10.1016/j.jbc.2021.100989

**Published:** 2021-07-21

**Authors:** Yuichi Watanabe, Takashi Sasaki, Shoko Miyoshi, Makoto Shimizu, Yoshio Yamauchi, Ryuichiro Sato

**Affiliations:** 1 Food Biochemistry Laboratory, Department of Applied Biological Chemistry, Graduate School of Agricultural and Life Sciences, University of Tokyo, Tokyo, Japan; 2 Nutri-Life Science Laboratory, Department of Applied Biological Chemistry, Graduate School of Agricultural and Life Sciences, University of Tokyo, Tokyo, Japan; 3 AMED-CREST, Japan Agency for Medical Research and Development, Tokyo, Japan

**Keywords:** sterol, endoplasmic reticulum stress, cell death, eukaryotic initiation factor 2, stress response, activating transcription factor-4, oxysterol, 25HC, 25-hydroxycholesterol, 27HC, 27-hydroxycholesterol, ATF4, activating transcription factor-4, CHO, Chinese hamster ovary, CHOP, C/EBP homology protein, eIF2α, eukaryotic translation initiation factor 2α, GCN2, general control nonderepressible 2, HMGCR, 3-hydroxy-3-methylglutaryl coenzyme A reductase, INSIG, insulin-induced gene, ISRIB, integrated stress response inhibitor, LXR, liver x receptor, MTT, 3-(4,5-dimethylthiazol-2-yl)-2,5-diphenyl-2H-tetrazolium bromide, ORP8, OSBP-related protein 8, OSBP, oxysterol-binding protein, PERK, protein kinase RNA-activated–like ER kinase, SCAP, SREBP cleavage–activating protein, SREBP, sterol regulatory element–binding protein

## Abstract

Insulin-induced genes (INSIGs) encode endoplasmic reticulum–resident proteins that regulate intracellular cholesterol metabolism. Oxysterols are oxygenated derivatives of cholesterol, some of which orchestrate lipid metabolism *via* interaction with INSIGs. Recently, it was reported that expression of activating transcription factor-4 (ATF4) was induced by certain oxysterols; the precise of mechanism is unclear. Herein, we show that INSIGs mediate ATF4 upregulation upon interaction with oxysterol. Oxysterols that possess a high affinity for INSIG, such as 27- and 25-hydroxycholesterol (25HC), markedly induced the increase of ATF4 protein when compared with other oxysterols. In addition, ATF4 upregulation by these oxysterols was attenuated in INSIG1/2-deficient Chinese hamster ovary cells and recovered by either INSIG1 or INSIG2 rescue. Mechanistic studies revealed that the binding of 25HC to INSIG is critical for increased ATF4 protein *via* activation of protein kinase RNA-activated–like ER kinase and eukaryotic translation initiation factor 2α. Knockout of INSIG1 or INSIG2 in human hepatoma Huh7 cells attenuated ATF4 protein upregulation, indicating that only one of the endogenous INSIGs, unlike overexpression of intrinsic INSIG1 or INSIG2, was insufficient for ATF4 induction. Furthermore, ATF4 proactively upregulated the cell death–inducible gene expression, such as *Chop*, *Chac1*, and *Trb3*, thereby markedly reducing cell viability with 25HC. These findings support a model whereby that INSIGs sense an increase in oxysterol in the endoplasmic reticulum and induce an increase of ATF4 protein *via* the protein kinase RNA-activated–like ER kinase–eukaryotic translation initiation factor 2α pathway, thereby promoting cell death.

Oxysterols are cholesterol metabolites produced through enzymatic or radical processes (1). Synthesized oxysterols are further metabolized to bile acids or steroid hormones (2). These oxysterols regulate sterol and lipid homeostasis as well as the immune system by acting as ligands for nuclear receptors or G protein–coupled receptors (3, 4, 5). Moreover, recent studies have revealed that oxysterols have a multifunction, such as contribution to protection against Zika virus or severe acute respiratory syndrome coronavirus 2 infection (6, 7). Meanwhile, certain oxysterols have also been reported as stressors that induce cytotoxicity or cell death and are implicated in various diseases (8, 9, 10, 11).

Insulin-induced genes (INSIGs), which consist of two isoforms (INSIG1 and INSIG2), are representative endoplasmic reticulum (ER)–resident oxysterol-binding proteins (OSBPs). Under the oxysterol-rich condition, INSIGs negatively regulate lipid and sterol synthesis through two distinct mechanisms. Sterol regulatory element–binding proteins (SREBPs) are activated by translocation from ER to Golgi *via* escorting by SREBP cleavage–activating protein (SCAP). Activated SREBPs regulate the expression of lipid and sterol synthetic genes. INSIGs bound with oxysterols negatively regulate the activation of SREBP by anchoring SCAP–SREBP complex to ER (12, 13). Besides, INSIGs proteolytically regulate cholesterol synthesis. INSIGs interact with 3-hydroxy-3-methylglutaryl coenzyme A reductase (HMGCR), the rate-limiting enzyme for cholesterol synthesis, and promotes its ubiquitination and degradation (14). Moreover, since the novel function of INSIGs, such as antiviral function, has recently been revealed, it is considered that INSIGs are essential for not only regulations of sterol and lipid homeostasis but also various signaling mediations (15).

Recently, it was reported that some oxysterols induce the expression of activating transcription factor-4 (ATF4) and its target genes (16, 17). ATF4 is a stress-inducible leucine zipper transcription factor that regulates cellular responses in order to adapt to various cellular stresses. Induction of ATF4 ordinarily aids cell survival and recovery through various regulations, such as genes involved in amino acid homeostasis, protection from oxidative stress, and protein homeostasis (18, 19). However, when the cellular stress becomes severe in intensity or duration, it turns to execute cell death (20). ATF4 is a central mediator of the integrated stress response and whose induction requires phosphorylation of eukaryotic translation initiation factor 2α (eIF2α), which is involved in the initiation step of protein biosynthesis (21). Under ER stress conditions, protein kinase RNA-activated–like ER kinase (PERK) is activated to phosphorylate eIF2α (22). Some reports suggested that oxysterols induce ER stress (17, 23); however, the precise of mechanism is still unclear.

In this study, we provide evidence that PERK activation mediated ATF4 induction and associated cell death by oxysterol. Besides, we also revealed that INSIGs–oxysterol interaction on ER membrane was required for inducing these cellular responses.

## Results

### ATF4 induction by oxysterols is decreased in INSIGs-deficient cell SRD-15

**Figure 1:**
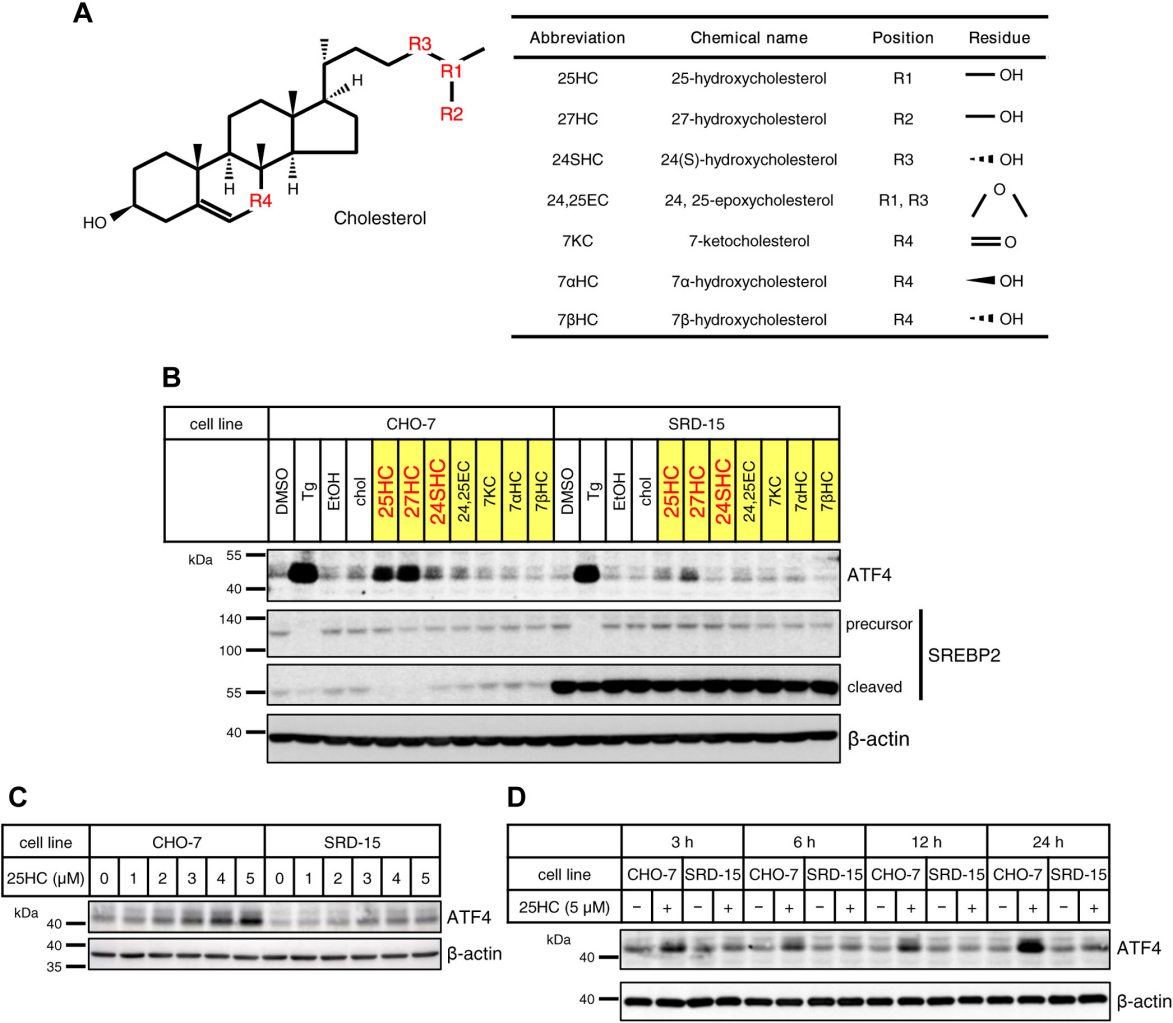
**INSIG expression is required for ATF4 induction by oxysterols.***A*, the *left panel* shows the chemical structure of cholesterol. The *right table* shows oxygenated sites and their functional groups in indicated oxysterols, which were used in this study. *B*, CHO-7 and SRD-15 cells were treated with 5 μM cholesterol (chol) or indicated oxysterols dissolved in ethanol for 24 h, or 250 nM thapsigargin (Tg) dissolved in DMSO for 3 h. *C*, CHO-7 and SRD-15 cells were treated with 25HC for 24 h at the indicated concentrations. *D*, CHO-7 and SRD-15 cells were treated with 5 μM 25HC for the indicated periods. The final concentration of ethanol or DMSO in the medium was 0.1%. *B*–*D*, whole-cell lysates were subjected to SDS-PAGE, followed by immunoblot analysis with specific antibodies as indicated. The quantified data obtained in the biological triplicate assay are shown in Fig. S1. 25HC, 25-hydroxycholesterol; ATF4, activating transcription factor-4; CHO-7, Chinese hamster ovary 7 cells; DMSO, dimethyl sulfoxide; INSIG, insulin-induced gene.

A previous article reported that 25-hydroxycholesterol (25HC), when added to the cell culture medium, causes stress in cells and increases ATF4 protein expression (17). Given that ER stress might initiate the PERK–ATF4 pathway, INSIG, an ER-resident membrane protein, appears to be one of the potential candidate proteins that sense a change in oxysterol levels and trigger ER stress. To verify this hypothesis, we utilized Chinese hamster ovary (CHO) mutant cells, SRD-15, which lack endogenous INSIG1 and INSIG2 (24). When parental CHO-7 and SRD-15 cells were incubated with an ER stress inducer, thapsigargin, a highly increased level of ATF4 protein was observed in both cells, suggesting that deficiency of INSIGs has no effects on PERK–ATF4 pathway (Fig. 1*B* and Fig. S1*A*). These cells were also incubated with seven types of oxysterols (Fig. 1*A*) with higher or lower INSIG-binding capacity for 24 h to clarify the need for INSIG to bind to oxysterols in order to elevate ATF4 protein levels. Two kinds of oxysterol, such as 25HC and 27-hydroxycholesterol (27HC), which were reported to possess a higher affinity for INSIG than other oxysterols (25), significantly increased ATF4 protein levels in CHO-7 cells, whereas these oxysterols only slightly raised ATF4 protein levels in SRD-15 cells (Fig. 1, *A* and *B* and Fig. S1*A*). These results suggest that these oxysterols induced an increased expression of ATF4 protein *via* interaction with INSIG as shown in the previous reports (25). As oxysterols are known to suppress SREBP processing by binding to INSIG, SREBP-2 activation was evaluated by immunoblot analysis using anti-SREBP-2 antibodies against its C-terminal region, which detect the precursor form of SREBP-2 in the ER and the cleaved form of SREBP-2 retained in the Golgi after the release of its N-terminal domain. In SRD-15 cells, a large amount of the cleaved form of SREBP-2 was detectable even in the presence of oxysterols because of lack of INSIGs, thus suggesting that the cleaved form was stably retained in the Golgi for a relatively long period. In the presence of thapsigargin, both the precursor and cleaved forms of SREBP-2 declined dramatically in these two cells probably because ER stress induced by thapsigargin severely suppressed the global translation of genes. Notably, both 25HC and 27HC appear to interact with INSIGs, thereby hindering SREBP-2 processing in CHO-7 cells; whereas, in SRD-15 cells, such effects are abolished because of lack of functional INSIGs. Unlike oxysterols, cholesterol itself is a very weak suppressor in terms of SREBP-2 processing. On the other hand, two oxysterols with enhanced ATF4 expression are also known as typical endogenous ligands for the liver x receptor (LXR) (26, 27). We, therefore, investigated whether the ATF4 protein level is increased also in CHO-7 cells cultured with a synthesized LXR agonist T0901317 for 24 h. While a significant increase in the ATF protein level was induced by 25HC, no increase was observed upon T0901317 (Fig. S2*A*). Regarding the LXR target genes such as *Abca1* and *Abcg1*, T0901317 more significantly increased their expression than 25HC (Fig. S2*B*). These results indicate no involvement of LXR in the increased ATF4 protein level induced by oxysterols. Next, CHO-7 and SRD-15 cells were treated with 25HC at different doses for 24 h or 5 μM 25HC for varying periods. In CHO-7 cells, 25HC increased ATF4 protein level in a dose-dependent manner, beginning with 2 μM, whereas ATF4 protein induction by 25HC in SRD-15 cells was attenuated when compared with that in CHO-7 cells (Fig. 1*C* and Fig. S1*B*). Besides, ATF4 protein in CHO-7 cells was induced by treating with 5 μM 25HC for 3 h and further increased after continuous culture with 25HC. Meanwhile, ATF4 protein induction by 25HC in SRD-15 cells was quite low even after 24 h incubation (Fig. 1*D* and Fig. S1*C*). Thus, induction of ATF4 protein by 25HC was mediated by INSIG in a dose- and time-dependent manner.

### INSIGs enhance ATF4 protein expression in a concentration-dependent manner by detecting 25HC

**Figure 2:**
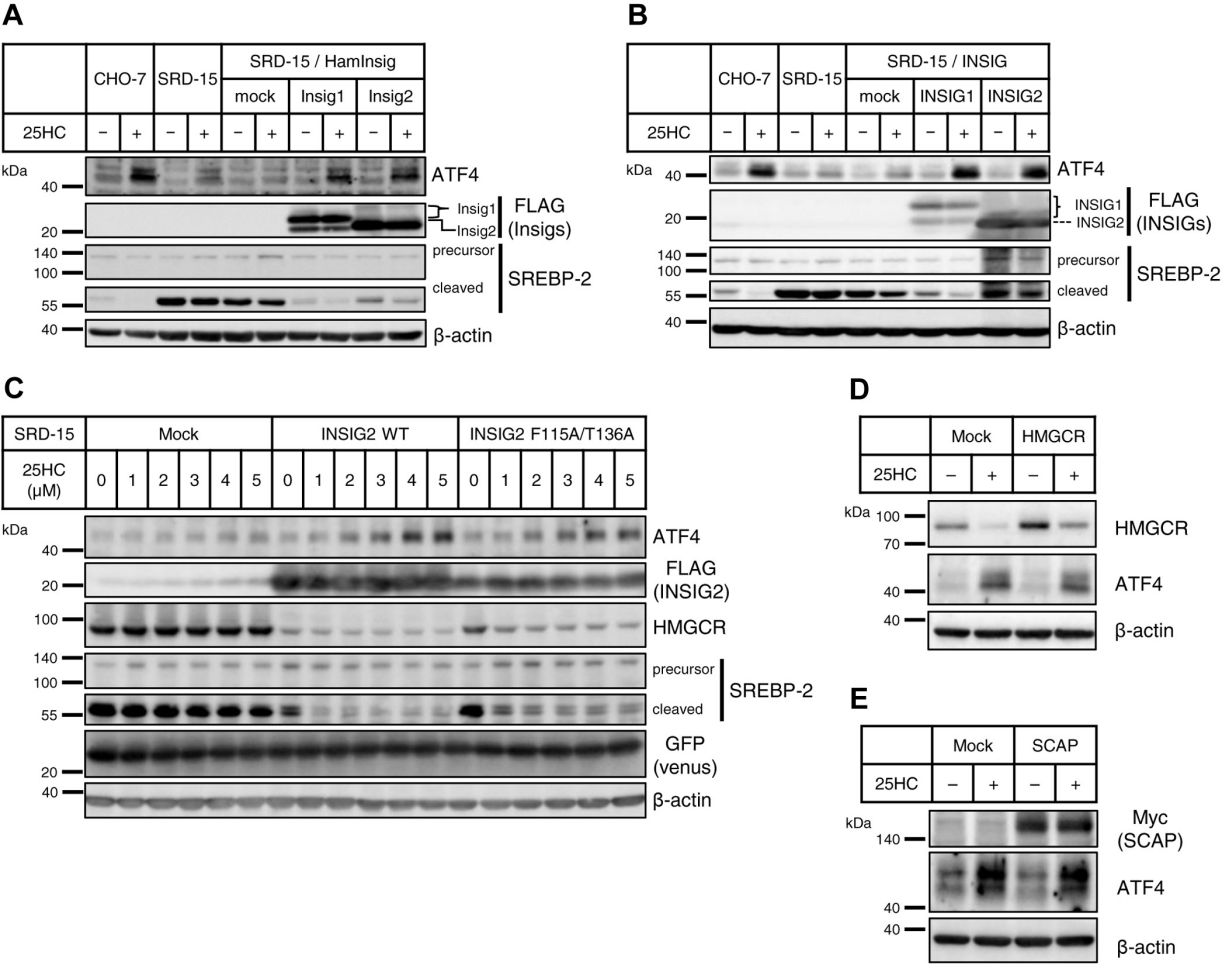
**INSIG bound with 25HC elevates ATF4 protein independently of its classical function.***A* and *B*, CHO-7 cells, SRD-15 cells, mock vector–infected SRD-15 cells, and FLAG-tagged Chinese hamster Insig1 or Insig2 (*A*) or FLAG-tagged human INISG1 or INSIG2 (*B*) overexpressed SRD-15 were treated with 5 μM 25HC for 24 h. *C*, mock vector–infected SRD-15 cells, and WT or mutated human INSIG2-FLAG overexpressed SRD-15 cells were treated with indicated concentrations of 25HC for 24 h. *D* and *E*, CHO-7 cells were transfected with pcDNA3.1 empty vector and His-HMGCR (*D*) or Myc-SCAP (*E*) expression plasmid. Each transfected cell was reseeded onto a 6-well plate and grown for 24 h. Then, cells were treated with 5 μM 25HC for 24 h. The final concentration of ethanol in the medium was 0.1%. Whole-cell lysates were subjected to SDS-PAGE, followed by immunoblot analysis with specific antibodies as indicated. The quantified data obtained in the biological triplicate assay were shown in Fig. S3. 25HC, 25-hydroxycholesterol; ATF4, activating transcription factor-4; CHO-7, Chinese hamster ovary 7 cells; INSIG, insulin-induced gene; SCAP, SREBP cleavage–activating protein.

As shown in Figure 1, *C* and *D*, it was shown that upregulation of ATF4 protein expression by 25HC treatment was attenuated in SRD-15 cells. To further implicate INSIGs in ATF4 induction by 25HC, SRD-15 cells were infected with a lentivirus vector containing Chinese hamster *Insig1* or *Insig2* complementary DNA. Responses of these infected cells treated with 25HC were analyzed by SDS-PAGE, followed by immunoblot analysis. In CHO-7 cells, treatment with 25HC reduced the protein level of cleaved SREBP2 caused by INSIG-mediated suppression of SREBP processing; whereas, in SRD-15 cells and SRD-15 cells infected with mock vectors, no decrease in the cleaved SREBP2 level was observed because SREBP2 activation was not suppressed because of the deficiency of INSIGs. Meanwhile, in SRD-15 cells expressing Chinese hamster INSIG1 or INSIG2, the protein level of cleaved SREBP2 was decreased in the presence of 25HC, thus suggesting that INSIGs were exogenously expressed and had proper functions. The ATF4 protein level, which increased in CHO-7 cells cultured with 25HC, but not in SRD-15 cells, was elevated in SRD-15 cells expressing either INSIG1 or INSIG2 (Fig. 2*A*). These results indicate that INSIGs are indispensable for increased ATF4 expression induced by 25HC.

In the functional analysis of INSIGs, such as oxysterol-binding ability and lipid metabolism control, human INSIG1 and INSIG2 were investigated. To clarify in detail the function and importance of INSIGs in oxysterol-induced ATF4 expression, further experiments using human INSIGs were performed. SRD-15 cells expressing FLAG-tagged human INSIG1 or INSIG2, which were prepared as shown in Figure 2*A*, were cultured with 5 μM 25HC. Enforced expression of human INSIG1 or INSIG2 reduced the cleaved SREBP2 levels in the presence of 25HC, thereby ensuring the appropriate function of these OSBPs (Fig. 2*B*). ATF4 protein level was increased by 25HC treatment in cells expressing INSIG1 or INSIG2, as in the case of expressing Chinese hamster INSIGs.

INSIG is a typical binding protein of oxysterols, and its function is evoked by binding to oxysterols. As shown in Figure 1*B*, two types of highly INSIG-binding oxysterols (25HC and 27HC) indeed enhanced ATF4 protein expression. This finding led us to speculate that the oxysterol binding to INSIG induces an increase of ATF4 protein. It has been reported that human mutant INSIG2, at Phe115 or Thr136, reduces the ability to bind oxysterols (25). Based on this finding, we established SRD-15 cells expressing double mutant INSIG2 with Ala residues at Phe115 and Thr136 separately from INSIG2 WT–expressing SRD-15 cells. These cells were cultured with the indicated concentrations of 25HC, and HMGCR and SREBP2 were detected by immunoblotting analysis. A certain level of venus protein detected in all the lanes shows that these cells were infected with an almost equal amount of exogenous genes. HMGCR, which was quite stable in SRD-15 cells infected with the mock vector, was reduced by the expression of INSIG2 WT in a 25HC concentration-dependent manner. In the mock cells, SREBP2 processing was not suppressed even when 25HC was raised to a concentration of 5 μM, whereas SREBP2 cleavage was attenuated by addition of 1 μM 25HC in the INSIG2 WT cells. On the other hand, INSIG2 F115A/T136A moderately promoted a decrease in HMGCR protein and an increase in 25HC concentration as compared with INSIG2 WT. Similarly, the mutant INSIG2 restored the regulated SREBP2 cleavage in the presence of 25HC, but modestly, as compared with INSIG2 WT. These results show that the F115A/T136A mutant version of INSIG2 partially missed INSIG functionality because of reduced oxysterol-binding capacity. When it comes to an increase in ATF4 expression in the presence of 25HC, INSIG2 WT robustly increased ATF4 protein with 25HC above 2 μM, but INSIG2 F115A/T136A only slightly increased ATF4 protein (Fig. 2*C*). From these results, it was suggested that the binding of oxysterol to INSIG plays an important role in the elevation of ATF4 expression.

The binding of oxysterol to INSIGs causes INSIGs to interact with HMGCR and SCAP, which respectively causes the degradation of HMGCR and suppression of SREBP2 activity (12, 13, 28, 29). In this regard, we investigated the influence of interaction between INSIGs and these associated proteins on INSIG-mediated increase in ATF4 protein. As shown in Figure 2*D*, in CHO-7 cells transfected with mock vector, a remarkable reduction of HMGCR protein was observed by incubation with 25HC. An enforced expression of HMGCR increased its basal protein level in CHO-7 cells, which was reduced in the presence of 25HC to the same level as that of mock cells incubated without 25HC. ATF4 protein increased in the presence of 25HC and also in HMGCR-overexpressing cells (Fig. 2*D* and Fig. S3*D*), thus suggesting no negative effect of the excess amount of HMGCR on INSIG-mediated ATF4 induction. Conversely, even though endogenous HMGCR expression was suppressed with siRNA specific for HMGCR in CHO-7 cells, which mimicked the situation cultured with 25HC, no remarkable ATF4 increase was observed (Fig. S4*A*). Moreover, overexpression of another INSIG-binding protein SCAP did not affect INSIG-mediated ATF4 induction (Fig. 2*E* and Fig. S3*E*). Since SCAP act as an escort protein for translocating SREBP from ER to Golgi, the depletion of SCAP causes suppression of SREBP activity (30). Although, in SRD-15 cells, SREBP2 is constitutively cleaved and activated because of lack of INSIGs, SCAP knockdown suppressed the SREBP2 cleavage, which mimicked the situation where 25HC hampered SREBP2 processing in CHO-7 cells (Fig. S4*B*). An increase in ATF4 protein was observed in 25HC-treated CHO-7 cells but not in SCAP-depleted SRD-15 cells. Taken together, INSIG-binding proteins, HMGCR and SCAP, do not affect the upregulation of ATF4 protein driven by INSIG–25HC interaction.

### 25HC-induced ATF4 selectively elevates cell death–inducible gene expression to cause cell death

**Figure 3:**
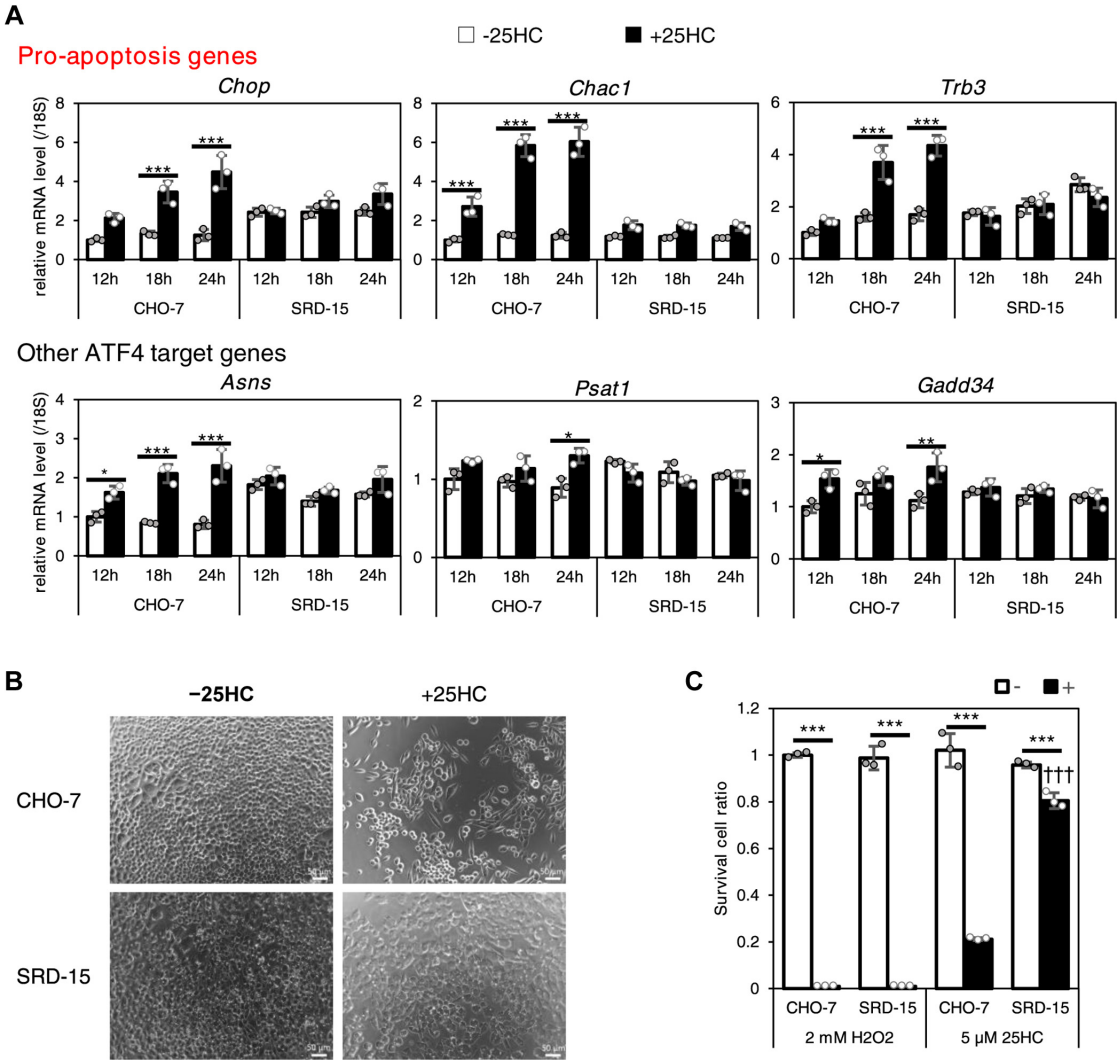
**INSIG deletion attenuates selective upregulation of cell death–inducible ATF4 target genes and restores the decline in cell viability by 25HC.***A*, CHO-7 and SRD-15 cells were treated with 5 μM 25HC for the indicated time. The mRNA of ATF4 target genes was measured using appropriate primers by RT-quantitative PCR. *B* and *C*, CHO-7 and SRD-15 cells were treated with 5 μM 25HC for 48 h (*B* and *C*) or with 2 mM H_2_O_2_ for 48 h (*C*). Representative images showing the cellular morphology were observed by microscope (*B*), and the cell viabilities were measured by MTT assay (*C*). The scale bar indicates 50 μm. The final concentration of ethanol in the medium was 0.1%. The data show mean ± SD obtained in the biological triplicate assay. ∗*p* < 0.05, ∗∗*p* < 0.01, and ∗∗∗*p* < 0.001 (relative to −25HC), or ^†††^*p* < 0.001 (relative to CHO-7). 25HC, 25-hydroxycholesterol; ATF4, activating transcription factor-4; CHO-7, Chinese hamster ovary cells; INSIG, insulin-induced gene; MTT, 3-(4,5-dimethylthiazol-2-yl)-2,5-diphenyl-2H-tetrazolium bromide.

ATF4, a stress-inducible transcription factor, promotes either cell survival or cell death, depending on the intensity and duration of stimulation. To examine the influence of increased INSIG-mediated ATF4 expression by oxysterols, a follow-up survey of alteration of ATF4 target gene expression was conducted up to 24 h in the presence of 25HC. In CHO-7 cells, ATF4 protein level was increased by 25HC in a time-dependent manner (Fig. 1*D*). Accompanying this increase is the expression of cell death–inducible ATF4 target genes *Chop*, *Chac1*, and *Trb3*, which increased from fourfold to sixfold in 24 h, whereas no increase was observed in SRD-15 cells. In contrast, *Asns* and *Psat1*, which are responsible for amino acid synthesis, or *Gadd34*, another ATF4 target gene that eventually downregulates ATF4 gene expression, showed little or no expression change by 25HC in both CHO-7 and SRD-15 cells (Fig. 3*A*). These results suggest that INSIG-mediated increase in ATF4 protein by 25HC might cause cell death by selectively upregulating cell death–inducible genes. Indeed, a multitude of CHO-7 cells shrank after 48 h culture with 25HC, whereas no morphological changes were observed in SRD-15 cells under the same conditions in comparison with the untreated cells (Fig. 3*B*). Cell viability of CHO-7 and SRD-15 cells after 48 h incubation with 5 μM 25HC was quantitatively determined by the 3-(4,5-dimethylthiazol-2-yl)-2,5-diphenyl-2H-tetrazolium bromide (MTT) assay using MTT. Incubation with 2 mM H_2_O_2_ for 48 h resulted in a marked decrease in cell viability in both groups. On the other hand, the cell viability with 5 μM 25HC was reduced to 20% of the control in CHO-7 cells; whereas, in SRD-15 cells, nearly 80% of the cells survived even in the presence of 25HC (Fig. 3*C*). These results suggest that INSIG-mediated upregulation of ATF4 by oxysterols selectively promotes cell death–inducible gene expression and leads to cell death.

### 25HC–INSIG–ATF4 axis is conserved in Huh7 cells

**Figure 4:**
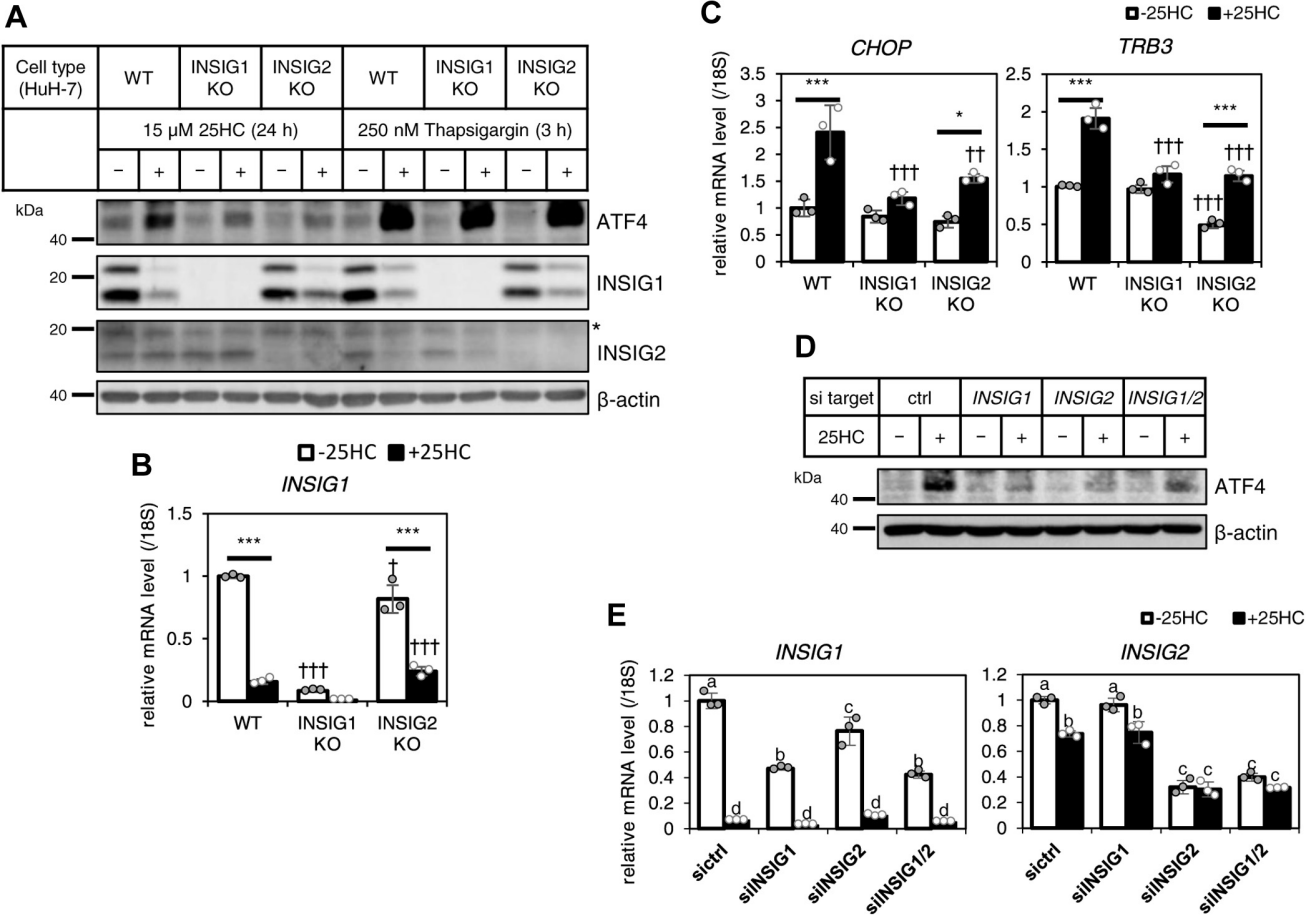
**INSIG depletion in Huh7 cells also reduces 25HC-induced ATF4 protein level and its target gene level.***A*–*C*, WT, INSIG1-deficient (INSIG1 KO), or INSIG2-deficient (INSIG2 KO) Huh7 cells were treated with 15 μM 25HC for 24 h (*A*–*C*), or 250 nM thapsigargin for 3 h (*A*). Whole-cell lysates were subjected to SDS-PAGE, followed by immunoblot analysis with specific antibodies as indicated (*A*). The *asterisk* in panel *A* indicates a nonspecific band. The mRNA of *INSIG1* (*B*) or ATF4 target genes (*C*) was measured by RT-quantitative PCR using appropriate primers. *D* and *E*, Huh7 cells transfected with control (ctrl) or *INSIG1* and/or *INSIG2* targeting siRNA were treated with 15 μM 25HC for 24 h. Whole-cell lysates were subjected to SDS-PAGE, followed by immunoblot analysis with specific antibodies as indicated (*D*). The mRNA expressions of *INSIG1* or *INSIG2* were measured using appropriate primer by RT-quantitative PCR (*E*). The final concentration of ethanol or DMSO in the medium was 0.3%. The data show mean ± SD obtained in the triplicate assay. ∗*p* < 0.05, ∗∗*p* < 0.01, and ∗∗∗*p* < 0.001 (relative to −25HC), ^†^*p* < 0.05, ^††^*p* < 0.01, and ^†††^*p* < 0.001 (relative to CHO-7) (*B* and *C*), or different *lower-case letters* indicate significant differences (*p* < 0.05) (*E*). 25HC, 25-hydroxycholesterol; ATF4, activating transcription factor-4; CHO-7, Chinese hamster ovary 7 cells; DMSO, dimethyl sulfoxide; INSIG, insulin-induced gene.

To investigate that the importance of INSIGs in ATF4 induction by 25HC is conserved across cell types and species, INSIG1- or INSIG2-deficient human hepatocarcinoma Huh7 cells were established using CRISPR–Cas9 system. The ATF4 protein levels increased in both INSIG1- and INSIG2-deficient Huh7 cells at the same level in parental WT Huh7 cells after 3 h incubation with 250 nM thapsigargin. This result suggests that INSIG deficiency did not affect the PERK–ATF4 pathway, as shown in SRD-15 cells in Figure 1*B*. The ATF4 upregulation in response to 25HC observed in the WT Huh7 cells was attenuated in both INSIG1- and INSIG2-deficient cells (Fig. 4*A*). The INSIG1 protein level was significantly decreased by 25HC in the WT and INSIG2-deficient Huh7 cells. Since *INSIG1* is one of the SREBP target genes, *INSIG1* mRNA levels in three types of Huh7 cells in the presence or the absence of 25HC were quantified (Fig. 4*B*). The result supports the notion that the lowered transcription of *INSIG1* gene caused by SREBP inactivation in response to 25HC led to the decreased INSIG1 protein level in the WT and INSIG2-deficient Huh7 cells. Reflecting the pattern of ATF4 protein levels, the increased C/EBP homology protein (*CHOP*) and *TRB3* mRNA levels by 25HC in WT Huh7 cells were significantly attenuated in INSIG-deficient cells (Fig. 4*C*). Next, using gene-specific siRNAs, INSIG1, INSIG2, and INSIG1/2 double knockdown cells were prepared. In these cells, INSIG1 gene expression was suppressed by almost 60%, whereas INSIG2 gene expression was suppressed by almost 70% (Fig. 4*E*). The increase in ATF4 protein in response to 25HC was attenuated by single and double knockdown of INSIG1 and INSIG2 (Fig. 4*D*). The result indicates that INSIG1 and INSIG2 almost equally serve as a 25HC sensor to induce an increased expression of ATF4 protein and that partial deficiency of one of them was quite sufficient to stem the signal transduction of the 25HC–INSIG–ATF4 pathway. Taken together, oxysterols increased ATF4 protein levels *via* INSIGs, followed by induction of a stress response in human hepatocarcinoma.

### ATF4 expression by 25HC–INSIGs is triggered *via* PERK pathway

**Figure 5:**
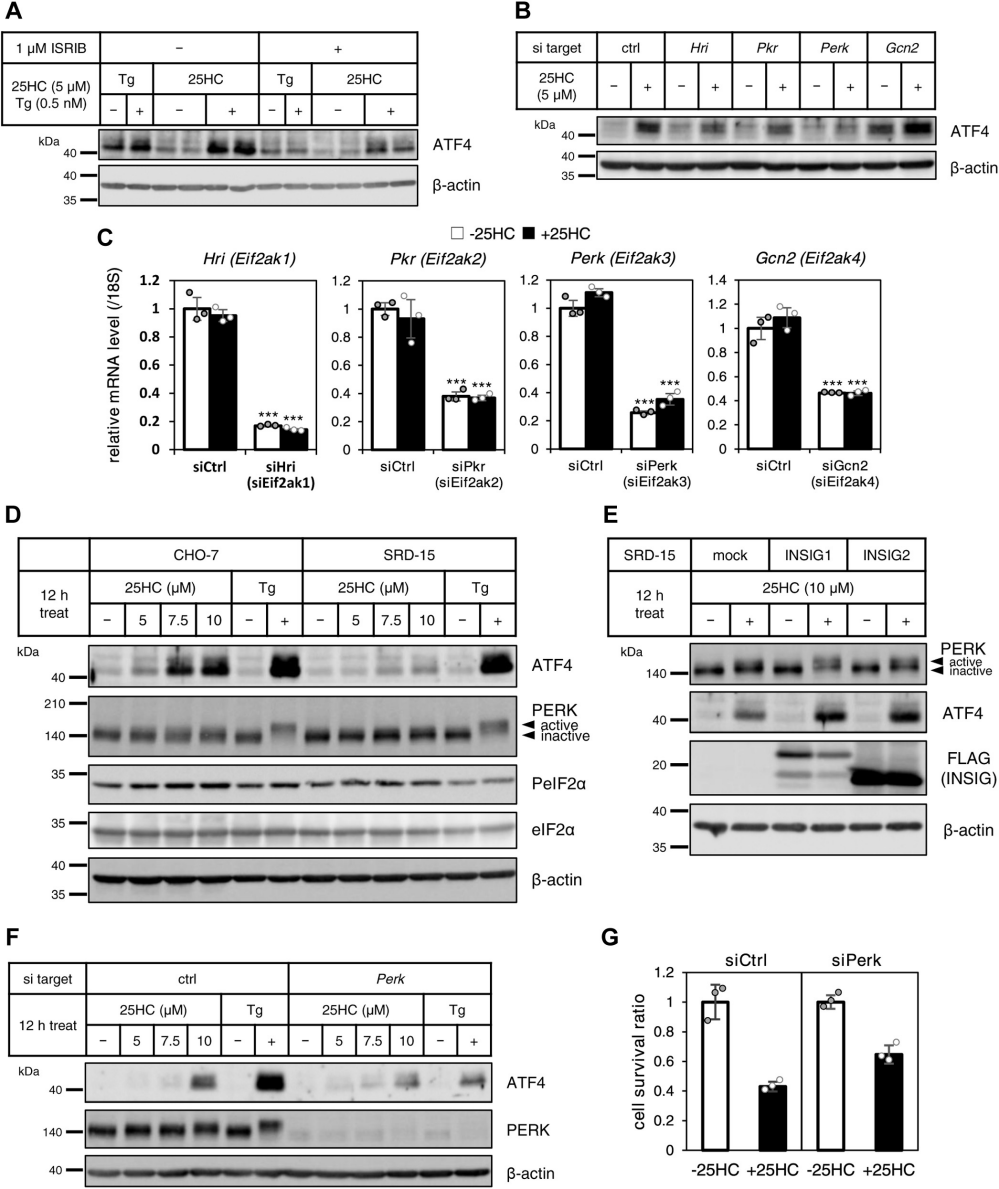
**25HC elicits activation of PERK *via* INSIG causing upregulation of eIF2α–ATF4 pathway, followed by cell death.***A*, CHO-7 cells were pretreated with 1 μM ISRIB for 30 min and then treated with 5 μM 25HC or 0.5 nM thapsigargin in the presence or the absence of 1 μM ISRIB for 24 h. The final concentrations of ethanol and DMSO in the medium were 0.1% each. *B* and *C*, CHO-7 cells transfected with control (ctrl), *Hri*, *Pkr*, *Perk*, or *Gcn2* targeting siRNA were treated with 5 μM 25HC for 24 h. The final concentration of ethanol in the medium was 0.1%. *D*, CHO-7 and SRD-15 cells were treated with indicated concentrations of 25HC or 100 nM thapsigargin for 12 h. *E*, mock vector–infected SRD-15 cells, and FLAG-tagged human INISG1, or INSIG2 overexpressed SRD-15 cells were treated with 10 μM 25HC for 12 h. *F*, CHO-7 transfected with control (ctrl) or *Perk* targeting siRNA were treated with indicated concentrations of 25HC and 100 nM thapsigargin for 12 h. The final concentration of ethanol or DMSO was 0.2 or 0.1%, respectively. Whole-cell lysates were subjected to SDS-PAGE, followed by immunoblot analysis with specific antibodies as indicated (*A*, *B*, and *D*–*F*). The mRNA expressions of each siRNA target were measured using appropriate primers by RT-quantitative PCR (*C*). *G*, CHO-7 transfected with control (ctrl) or *Perk* targeting siRNA were treated with 5 μM 25HC for 48 h. The cell viabilities were measured by MTT assay. The final concentration of ethanol in the medium was 0.1%. The data show mean ± SD obtained in the triplicate assay. ∗*p* < 0.05, ∗∗*p* < 0.01, and ∗∗∗*p* < 0.001 (relative to siCtrl). 25HC, 25-hydroxycholesterol; ATF4, activating transcription factor-4; CHO-7, Chinese hamster ovary 7 cells; DMSO, dimethyl sulfoxide; eIF2α, eukaryotic translation initiation factor 2α; INSIG, insulin-induced gene; ISRIB, integrated stress response inhibitor; MTT, 3-(4,5-dimethylthiazol-2-yl)-2,5-diphenyl-2H-tetrazolium bromide; PERK, protein kinase RNA-activated–like ER kinase.

Phosphorylation of eIF2α leads to suppression of global protein translation and preferential translation of ATF4 independently of its transcription. The integrated stress response inhibitor (ISRIB) suppresses ATF4 translation by rendering cells insensitive to this phosphorylation. To investigate whether oxysterol-derived ATF4 depends on the phosphorylation of eIF2α, the effect of ISRIB on upregulation of ATF4 by 25HC was analyzed. As shown in Figure 5*A*, induction of ATF4 by thapsigargin in CHO-7 cells was attenuated by 1 μM ISRIB. Similarly, upregulation of ATF4 by 25HC was attenuated by ISRIB. This result suggested that induction of ATF4 by oxysterol is mediated by phosphorylation of eIF2α. Four types of enzymes, including heme-regulated inhibitor kinase, double-stranded RNA-activated protein kinase, PERK, and general control nonderepressible 2 (GCN2), are known as kinases that phosphorylate eIF2α in response to various stresses. To investigate which kinase is responsible for the phosphorylation of eIF2α by oxysterol, the impact of knockdown of each kinase using siRNAs to the induction of ATF4 by 25HC was assessed. As a result, mRNA levels of the kinases were suppressed to 20 to 40% by each siRNA. The 25HC-derived ATF4 upregulation was attenuated by knockdown of PERK among the four kinases assessed (Fig. 5, *B* and *C*). PERK, the ER membrane-resident eIF2α kinase, is activated by autophosphorylation and initiates phosphorylation of eIF2α under ER stress conditions. PERK activation, which was assessed by mobility shift, and marked ATF4 expression were confirmed in both CHO-7 and SRD-15 cells treated with 100 nM thapsigargin. Treatment of SRD-15 cells with 5 to 10 μM 25HC showed little upregulation of ATF4 and no mobility shift of PERK. On the other hand, in CHO-7 cells cultured with 10 μM 25HC, a slight activation of PERK was confirmed, accompanied by the upregulation of ATF4 expression. Furthermore, a 25HC concentration-dependent increase in phosphorylation of eIF2α was confirmed in CHO-7 cells but not in SRD-15 cells (Fig. 5*D*). Enforced expression of INSIG1 or INSIG2 in SRD-15 cells enhanced ATF4 upregulation and PERK activation by 25HC when compared with mock-infected SRD-15 cells (Fig. 5*E*). In mock-infected SRD-15 cells, a slight mobility shift of PERK was observed in the presence of 25HC and, as a result, ATF4 protein was also slightly increased (Fig. 5*E*, lanes 1 and 2). To clarify the involvement of PERK in ATF4 expression by 25HC, PERK-depleted CHO-7 cells (obtained *via* knockdown with siRNA) were incubated with 25HC. The expression of PERK protein was undetectable by PERK knockdown. In the control siRNA group, 100 nM thapsigargin and 10 μM 25HC upregulated ATF4 expression and PERK activation. On the other hand, PERK knockdown markedly suppressed the upregulation of ATF4 by either thapsigargin or 25HC (Fig. 5*F*). The attenuation of 25HC-induced ATF4 caused by PERK knockdown ameliorated the decrease in cell viability by 25HC. In control siRNA group, the cell viability was reduced to about 40% by 25HC; whereas, in PERK knockdown group, the cell viability by 25HC treatment was about 60% (Fig. 5*G*). From these results, it was suggested that INSIG-mediated upregulation of ATF4 by 25HC was mainly mediated by PERK pathway.

## Discussion

Oxysterols, which are synthetic intermediates of bile acids or steroid hormones, act as ligands for nuclear receptors or G protein–coupled receptors and maintain cellular homeostasis (31). Meanwhile, it was reported that the accumulation of oxysterols implicates in various diseases, such as arteriosclerosis and cancer, among others (32, 33, 34). Recently, oxysterol has been reported to upregulate the stress-inducible transcription factor ATF4 (16, 17). In this report, we show that INSIG, an ER-resident regulator of sterol and lipid metabolism, contributes to oxysterol-induced ATF4 upregulation and results in the induction of cell death.

First, we identified certain two oxysterols (25HC and 27HC) that markedly induced ATF4 compared with other oxysterols (Fig. 1*B*). These two oxysterols are known as ligands of LXR (26, 27), but the addition of T0901317, which enhanced the expression of LXR target genes more strongly than 25HC, did not increase the expression of ATF4. It was considered that LXR was not involved in the upregulation of ATF4 expression by oxysterols (Fig. S2). According to the previous report showing that both 25HC and 27HC possess a high affinity to INSIG (25), it is likely that they induced increased ATF4 protein by binding to INSIG. Indeed, the following findings further support the notion: (1) the induction of ATF4 protein by 25HC disappeared in INSIG-deficient CHO-7 and Huh7 cells (Figs. 1 and 4, *A* and *D*). (2) Separately enforced expression of INSIGs in SRD-15 cells, INSIG-deficient CHO-7 cells, restored ATF4 induction by 25HC (Fig. 2, *A* and *B* and Fig. S3, *A* and *B*). (3) The deletion of either isoform of two INSIGs led to an attenuated increase in ATF4 protein caused by 25HC in Huh7 cells (Fig. 4, *A* and *D*). In Huh7 cells, deficiency of either INSIG1 or INSIG2 attenuated ATF4 induction; whereas, in SRD-15 cells, enforced expression of either INSIG1 or INSIG2 restored ATF4 induction (Fig. 2, *A* and *B* and Fig. S3, *A* and *B*). Extrinsic INSIG1 or INSIG2 overexpressed in SRD-15 cells was sufficient to sense the increase of 25HC; whereas, in Huh7 cells deficient in either endogenous INSIG1 or INSIG2, the remaining INSIG might have been insufficient to capture 25HC for the sake of inducing an increased ATF4 protein. Since INSIGs are stabilized by binding with oxysterols, it seems natural that their function is initiated by interaction with oxysterols. A previous study demonstrated that two amino acid residues in INSIG2 (Phe115 and Thr136) are critical for the interaction with oxysterols and that those substitutions to Ala lower the 25HC-mediated inhibition of SREBP-2 processing (25). When the double mutant version of INSIG2 was expressed in SRD-5 cells, induction of ATF4 protein by 25HC was modest as compared with the WT INSIG2, thus indicating the importance of interaction between INSIG and oxysterols for transmitting a stress signal caused by the accumulation of oxysterols (Fig. 2*C* and Fig. S3*C*). Although we assessed the impact of amino acid substitution only in human INSIG2, the mutant INSIG1 with the same amino acid substitution ought to have given us a similar result. Human INSIG1 and INSIG2 share high amino acid sequence homology (84.65%) in their transmembrane domains and the amino acid sequences in the vicinity of Phe115 and Thr136 in INSIG2 are completely conserved in INSIG1 (Fig. S5). Taken together, it seems likely that INSIG1 and INSIG2 evenly induce an increased expression of ATF4 protein by binding to oxysterols.

INSIGs are prompted to interact with HMGCR and SCAP by binding with oxysterols, thereby regulating sterol and lipid metabolisms. When these associated proteins were overexpressed in CHO-7 cells, an increase in ATF4 protein in response to 25HC was not affected (Fig. 2, *D* and *E*). These results indicate that INSIGs maintain their effective function as an ATF4 inducer even in the presence of an excess amount of the interacting proteins, HMGCR and SCAP. Furthermore, knockdown of HMGCR in CHO-7 cells or SCAP in SRD-15 cells, which respectively mimicked HMGCR degradation or suppression of SREBP activation by oxysterol, did not affect ATF4 upregulation, which is consistent with a previous report (16) (Fig. S4, *A* and *B*). Taken together, INSIGs elevate ATF4 protein independently of the control of sterol and lipid metabolism.

ATF4 is known as a transcription factor that produces dual-opposite consequence, cell survival, and cell death. Asparagine synthetase and phosphoserine aminotransferase 1 induced by ATF4 make cells survive by controlling cellular amino acid metabolism (35, 36, 37) and GADD34, a phosphatase of eIF2α, negatively control the induction of ATF4 (38). On the other hand, CHOP, CHAC1, and TRB3 are cell death–inducible factors; and besides, the expression of CHOP triggers apoptosis under ER stress condition (39, 40). Selective induction of *Chop*, *Chac1*, and *Trb3* gene expression rather than genes related to amino acid metabolism alludes the notion that 25HC might elicit cell death in CHO-7 cells but not in SRD-15 cells (Fig. 3*A*). Indeed, the MTT assay with 25HC-treated CHO-7 and SRD-15 cells revealed that a long-term culture with 25HC significantly reduced the viability of CHO-7 cells, but only slightly affected the viability of SRD-15 cells (Fig. 3*C*). These results indicate that 25HC-induced ATF4 through INSIGs, which selectively triggers cell death by upregulating cell death–inducible factors.

The induction mechanism of ATF4 protein has been fully studied: phosphorylated eIF2α inhibits global protein synthesis and promotes selective translation of ATF4, which contains multiple short upstream open reading frames in its 5′ untranslated region (41, 42). The reduction of increased ATF4 protein with 25HC caused by ISRIB, an eIF2α inhibitor, supports the notion that this induction is likely to be initiated by eIF2α phosphorylation (Fig. 5*A*). Furthermore, our results on PERK knockdown indicate that the induction of ATF4 by 25HC is triggered by PERK activation (Fig. 5, *B* and *F*), which is in line with previous reports showing ER stress response stimulated by 25HC (17, 23). In contrast, Shibata *et al.* (16) reported that 25HC initiated an integrated stress response through GCN2 rather than PERK. As for this discordance, different cell types, such as macrophages and CHO cells, used in both experiments might make a difference in the activated pathway. PERK and GCN2 are thought to have redundancy in response to various stresses, and the main response pathway to ER stress may differ depending on the cell type (20, 43). In SRD-15 cells, the activation of PERK by 25HC diminished (Fig. 5*D*) and was restored by enforced expression of either INSIG1 or INSIG2 (Fig. 5*E*). Moreover, PERK knockdown in CHO-7 cells lowered cell death caused by 25HC (Fig. 5*G*). Taken together, the binding of oxysterol to INSIGs elicits activation of PERK in CHO and hepatoma cells, thereby upregulating the eIF2α–ATF4 pathway, followed by induction of cell death. To investigate the possibility of activation of PERK by direct binding to INSIGs, we performed immunoprecipitation experiments with a FLAG antibody using SRD-15 cells expressing either FLAG-tagged INSIG1 or INSIG2 and found no coimmunoprecipitates of INSIG–PERK (data not shown). As Ras homolog enriched in brain and protein disulfide isomerase have been reported to activate PERK (44, 45), INSIGs might crosstalk with any of these regulators.

A previous study reported that 25HC elevated ATF4 protein with the help of OSBP-related protein 8 (ORP8) in Huh7 cells (17). ORP8 is an ER-resident protein possessing oxysterol affinity through its OSBP-related ligand-binding domain (46). ORP8 is thought to enhance the accumulation of 25HC to ER membrane, which accelerates ER stress response. Thus, INSIGs might induce an increased expression of ATF4 protein by binding with oxysterol recruited by ORP8.

In conclusion, we herein provide a novel function of INSIG that mediates oxysterol-induced cell death. INSIGs sensing of an increase in oxysterol in the ER induce an increase of ATF4 protein through activation of PERK and subsequent phosphorylation of eIF2α. Increased ATF4 proactively upregulates the expression of cell death–inducible genes, eventually promoting cell death. Further analytical studies are needed to determine whether cell death caused by locally accumulated oxysterols because of dyslipidemia exacerbates metabolic disorders.

## Experimental procedures

### Materials

25HC, 27HC, 24SHC, 24,25EC, 7βHC, and ISRIB were procured from Sigma–Aldrich. 7KC was procured from Santa Cruz Biotechnology. 7αHC was procured from Abcam. Thapsigargin was procured from Wako. The following antibodies were procured from commercial sources: anti-ATF4, anti-eIF2α, antiphosphorylated eIF2α, and anti-PERK (code: 11815, 9722, 9721, and 3192) from Cell Signaling Technology; anti-SREBP2 and anti-INSIG1 (code: sc-13552 and sc-390504) from Santa Cruz Biotechnology; anti-β-actin, anti-FLAG, and anti-c-Myc (code: A5441, F1804, and C3956) from Sigma–Aldrich; anti-GFP (code: ab6556) from Abcam; and peroxidase-conjugated antimouse IgG and peroxidase-conjugated anti-rabbit IgG (code: 715-035-151 and 111-035-144) from Jackson ImmunoResearch. Anti-INSIG2 rabbit polyclonal antibody was produced by Cosmo Bio with an epitope to amino acids 215 to 225 of human INSIG2. Anti-HMGCR mouse monoclonal antibody (clone A9) and anti-Chinese hamster SREBP2 mouse monoclonal antibody (clone 7D4) were generous gifts from Dr Ta-Yuan Chang (Geisel School of Medicine). All other chemicals of analytical grade were obtained from Sigma–Aldrich, Wako, or nacalai tesque.

### Cell culture

CHO-7 and SRD-15 cells were maintained in Dulbecco's modified Eagle's medium/Ham's F12 supplemented with 100 units/ml penicillin, 100 μg/ml streptomycin sulfate, and 10% (v/v) fetal bovine serum. Huh7 and human embryonic kidney 293T cells were maintained in Dulbecco's modified Eagle's medium supplemented with 100 units/ml penicillin, 100 μg/ml streptomycin sulfate, and 10% (v/v) fetal bovine serum. All cells were cultured in a humidified incubator at 37 °C and 5% CO_2_.

### Plasmid construction

DNA fragment encoding human *INSIG1* (NM_005542) or *INSIG2* (NM_016133) and Chinese hamster *Insig1* (NM_001244079) or *Insig2* (NM_001244078) were amplified by PCR with appropriate primer sets in Table S1 and cloned into a p3×FLAG-CMV-14 vector (Sigma) using EcoRI/XbaI or HindIII/BamHI and EcoRI/BamHI restriction enzyme sites to construct human INISG1-FLAG or INSIG2-FLAG and Chinese hamster INSIG1-FLAG- or INSIG2-FLAG–expressing plasmid, respectively. For mutation of the *Bam*HI site without missense, the aforementioned constructed-expressing plasmid was subjected to inverse PCR mutagenesis using KOD-Plus-Mutagenesis kit (TOYOBO) following the manufacturer's protocol. To construct FLAG-tagged human INSIG2 F115A/T136A mutant-expressing plasmid, INSIG2-FLAG–expressing plasmid was subjected to inverse PCR mutagenesis as described previously. DNA fragment encoding Chinese hamster *Hmgcr* (XM_027401680) was amplified by PCR using pCMV7Red as a template (47) with appropriate primer sets in Table S1 and cloned into pcDNA3.1/Hygro (+) vector (Thermo Fisher Scientific) using KpnI–NotI restriction enzyme sites to construct N-terminal His-tagged Chinese hamster HMGCR-expressing plasmid. Primer sequences are listed in Table S1. The expression plasmid for N-terminal Myc-tagged human SCAP was described in a previous report (48). Preannealed oligo duplex Insig1-gRNAs/Insig1-gRNAa, Insig2-gRNAups/Insig2-gRNAupa, and Insig2-gRNAdowns/Insig2-gRNAdowna were cloned into pSpCas9(BB)-2A-Puro (PX459) V2.0 vector using BbsI restriction enzyme site to construct PX459/Insig1, PX459/Insig2upstream, and PX459/Insig2downstream each. The sequences of oligos for the preannealing are listed in Table S2.

### Lentivirus preparation and transduction

DNA fragment encoding C-terminal FLAG-tagged human *INSIG1*, *INSIG2*, or *INSIG2-F115A/T136A*, and Chinese hamster *Insig1* or *Insig2* were amplified by PCR with appropriate primer sets in Table S1 and cloned into a lentiviral vector CSII-EF-MCS-IRES2-Venus (RIKEN; RDB04383) using NotI and BamHI restriction enzyme sites to construct lentivector. For virus packaging, human embryonic kidney 293T cells were seeded in 100-mm dishes at 2 × 10^6^ and grown overnight until 70% confluence was reached. The cells were transfected with INSIG-FLAG or empty lentivector along with packaging plasmids pCAG-HIVgp (RIKEN; RDB04394) encoding VSV-G protein and HIV-1 Gag and Pol and pCMV-VSV-G-RSV-Rev (RIKEN; RDB04393) encoding VSV-G protein and RSV Rev by calcium phosphate method. To increase viral protein expression, 10 μM forskolin was applied to the cells 12 h after transfection. The culture supernatant containing viral particles was harvested 48 h after transfection and filtered through 0.45-μm sterile disks (Advantec; 25AS045AS). To express INISIG-FLAG, SRD-15 cells were seeded onto 6-well plates at 1.0 × 10^5^ cells/well and transduced with INSIG-FLAG or control lentivirus in the presence of 10 μg/ml polybrene 24 h after seeding. The culture medium containing virus and polybrene was replaced by a fresh medium 24 h after transduction.

### INSIG knockout Huh7 cells generation using CRISPR–Cas9

INSIG1- or INSIG2-deficient Huh7 cells were generated as follows. On day 0, Huh7 cells were seeded onto a 60-mm dish at 5.0 × 10^5^. On day 1, cells were transfected with 1 μg/dish of PX459/Insig1 or 1 μg/dish of PX459/Insig2upstream and 1 μg/dish of PX459/Insig2downstream using Lipofectamine 3000 according to the manufacturer's protocol. On day 2, cells were reseeded onto 100-mm dishes. On day 3, cells were switched to 0.5 μg/ml of puromycin-containing medium. Fresh medium was added every 2 to 3 days after a week. In the last 1 week, a fresh medium containing 0.5 μg/ml of puromycin and 0.25 μM 25HC was added every 2 to 3 days until colonies were formed. After the colony formation, individual colonies were isolated using cloning cylinders.

### Immunoblot analysis

Cells were lysed in lysis buffer (50 mM Tris‒HCl, pH 8.0, 1 mM EDTA, 150 mM NaCl, 1% NP-40, and 0.25% sodium deoxycholate). The lysate was centrifuged at 18,000*g* for 5 min at 4 °C in order to remove cellular debris. The supernatants were adjusted to appropriate concentrations and treated with 6× Laemmli sample buffer (1 mM Tris‒HCl, pH 6.8, 30% glycerol, 10% SDS, 600 mM dithiothreitol, and 0.03% bromophenol blue). The protein samples were analyzed by SDS-PAGE followed by immunoblot analysis with indicated antibodies and visualized by Amersham Bioscience ECL Western blotting detection reagent (GE Healthcare Life Sciences) or Immobilon Western chemiluminescent HRP substrate (Merck Millipore).

### Real-time quantitative PCR

Total RNA from CHO-7, SRD-15, Huh7, and INSIG1- or INSIG2-deficient Huh7 cells were extracted using ISOGEN (Nippon Gene) and reverse transcribed using High-Capacity complementary DNA Reverse Transcription Kit (Thermo Fisher Scientific) according to the manufacturer's protocol. Real-time quantitative PCR was performed with Applied Biosystems StepOnePlus real-time PCR system (Thermo Fisher Scientific) using FastStart Universal SYBR Green Master (Roche Applied Science) according to the manufacturer's protocol. All relative mRNA expression levels were normalized with *18S*. Primer sequences are listed in Table S3.

### Cell treatments

Before indicated treatments, CHO-7 cells, CHO-7 cells transfected with some expression plasmids or siRNAs, SRD-15 cells, and indicated lentivirus infected SRD-15 cells were seeded unto a 6-well plate at 1.0 × 10^5^ cells/well on day 0. In another experiment, Huh7 cells, Huh7 cells transfected with some siRNAs, and INSIG1- or INSIG2-deficient Huh7 cells were seeded unto a 6-well plate at 3.0 × 10^5^ cells/well on day 0. On day 1, cells were cultured in a medium containing indicated concentrations and periods of 25HC, other oxysterols, or thapsigargin. In another experiment, cells were pretreated with 1 μM ISRIB for 30 min and then cultured for 24 h in the medium containing either 5 μM 25HC or 0.5 nM thapsigargin in the presence or the absence of 1 μM ISRIB or ISRIB alone. After the indicated incubations, cells were harvested for immunoblot analysis or RT-quantitative PCR.

### RNA interference

The double-stranded siRNAs targeting human *INSIG1* and *INSIG2* and their negative control siRNA were obtained from Santa Cruz Biotechnology (code: sc-44432 and sc-45781, and sc-47007). The Silencer Select siRNA targeting Chinese hamster *Hri* (XM_027413593), *Pkr* (NM_001328108), *Perk* (XM_016976355), and *Gcn2* (XM_027422213) and their negative control siRNA (code: 4390843) were obtained from Thermo Fisher Scientific. The sense and antisense of siRNAs used in this study are listed in Table S4. Transfection of siRNAs was performed using Lipofectamine RNAiMAX Reagent (Thermo Fisher Scientific) according to the manufacturer's protocol.

### Cell viability measurement by MTT assay

On day 0, CHO-7 and SRD-15 cells were seeded onto a 96-well plate at 2.5 × 10^3^ cells/well. On day 1, cells were switched to the identical medium and incubated for 48 h. In another experiment, on day 0, CHO-7 cells were seeded and transfected with negative control siRNA and siRNA targeting Chinese hamster *Perk* using Lipofectamine RNAiMAX Reagent onto a 96-well plate at 2.5 × 10^3^ cells/well. After 24 h incubation, cells have switched to the medium and incubated with or without 25HC for 48 h. After 48 h incubation, cell viability was determined using Colorimetric Cell Viability Kit IV (MTT) (PromoCell GmbH).

### Statistical analysis

Data were presented as mean ± SD, and significance through one-way analysis of variance with Tukey's post hoc test was determined, unless otherwise stated.

## Data availability

All data are contained within the article.

## Supporting information

This article contains supporting information.

## Conflict of interest

The authors declare that they have no conflicts of interest with the contents of this article.
