# Supplementary material for: Insulin-induced genes INSIG1 and INSIG2 mediate oxysterol-dependent activation of the PERK–eIF2α–ATF4 axis
Source: J Biol Chem. 2021 Jul 21;297(2):100989. doi: 10.1016/j.jbc.2021.100989 (PMC8363831; doi:10.1016/j.jbc.2021.100989)
Supplement: Supplemental Figures S1–S5 and Tables S1–S4 [file mmc1.pdf]

Supporting information

Insulin-induced genes INSIG1 and INSIG2 mediate oxysterol-dependent activation of the PERK/eIF2α/ATF4 axis

Yuichi Watanabe[1], Takashi Sasaki[1], Shoko Miyoshi[1], Makoto Shimizu[2], Yoshio Yamauchi[1] and Ryuichiro Sato[1–3]*

[1]Food Biochemistry Laboratory and [2]Nutri-Life Science Laboratory, Department of Applied Biological Chemistry, Graduate School of Agricultural and Life Sciences, University of Tokyo, 1-1-1 Yayoi, Tokyo 113-8657, Japan and [3]AMED-CREST, Japan Agency for Medical Research and Development, Tokyo 100-0004, Japan

---

Experimental procedure

Cell treatment

CHO-7 cells were seeded onto a 6-well plate at 1.0 × 10^5 cells/well on day 0. On day 1, cells were cultured for 24 h in a medium containing indicated concentrations of 25HC or T0901317 (Cayman Chemical, Ann Arbor, MI). After the incubation, cells were harvested for immunoblot analysis or RT-qPCR.

Table S1 Sequences of PCR primers for cloning in expression vectors

PCR primers for cloning	Sequences (5’ to 3’)
**Cloning into pCMV14 primers**	
humanINSIG1-EcoRI-Fw	AAATTTATAGAATCCATGCCCAGATTGCACGACC
humanINSIG1-XbaI-Rv	AAATTTATATCTAGAATCACTATGGGGCTTTTCAGGAAC
	underline shows *Eco*RI and *Xba*I site, respectively
humanINSIG2-HindII-Fw	TTTATAAAGCTTATGGCAGAAGGAGAGACAG
humanINSIG2-BamHI-Rv	TAATGGATCCTTCCTGATGAGATTTTTCTGC
	underline shows *Hind*III and *Bam*HI site, respectively
hamsterInsig1-EcoRI-Fw	AAATTTATAGAATTCATGCCCAGGCTGCACGAC
hamsterInsig1-BamHI-Rv	TATAAATTTGGATCCGTCACTGTGAGGCTTTTCCGGA
	underline shows *Eco*RI and *Bam*HI site, respectively
hamsterInsig2-EcoRI-Fw	AAATTTATAGAATTCATGGCAGAAGGAAAGACCGAGT
hamsterInsig2-BamHI-Rv	TATAAATTTGGATCCTTCTTGATGAGATTTTTCAGGAATAACTTT
	underline shows *Eco*RI and *Bam*HI site, respectively
***Bam*HI mutation primers**	
INSIG-FLAG-BamHImut-Fw	ACGGGCTGACTACAAAGACCATGA
humanInsig1-FLAG-BamHImut-Rv	GATCCATCACTATGGGGCTTTT
humanInsig2-FLAG-BamHImut-Rv	GATCCTTCCTGATGAGATTTTTCTG
hamsterInsig1-FLAG-BamHImut-Rv	GATCCGTCACTGTGAGGCTTTTC
hamsterInsig2-FLAG-BamHImut-Rv	GATCCTTCTTGATGAGATTTTTCAGG
**INSIG2 F115A/T136A mutation primers**	
INSIG2-F115A-mut-Fw	CTGTTGGTATAAATCATGCCAGTGC
INSIG2-F115A-mut-Rv	CGACTGCTACACACCGCATTACACT
INSIG2-T136A-mut-Fw	CTCTGGCTGCACTATCCATTGG
INSIG2-T136A-mut-Rv	CGAGAGACAACTGTATGTTGTTATCGAAA

**Subcloning into lentivirus vector primers**	
humanINSIG1-FLAG-NotI-Fw	TACGGCGGCCGCATGCCCAGATTGCACGACC
humanINSIG2-FLAG-NotI-Fw	TACGGCGGCCGCATGGCAGAAGGAGAGACAGAGTCA
hamsterINSIG1-FLAG-NotI-Fw	AAATTTATAGCGGCCGCGAATTCATGC
hamsterINSIG2-FLAG-NotI-Fw	AAATTTATAGCGGCCGCGAATTCATGG
Insig-FLAG-BamHI-Rv	TAATGGATCCTCACTACTTGTCATCGTCATCCTTGTAG
	underline shows *Not*I or *Bam*HI site each
**Subcloning into pcDNA3.1/Hygro(+) vector primers**	
RGSHis-CHOHmgcr-Kpn-Fw	ATAGGTACCATGAGAGGTTCACACCATCACCATCACCATGAT ATCTTGTCACGACTTTTCCGTATGC
RGSHis-CHOHmgcr-Not-Rv	AAATTTATAGCGGCCGCTCAAGCTGACTTCTTGGTGCAC
	underline shows *Kpn*I or *Not*I site each

Table S2 Sequences of oligos for cloning in Cas9 vector

Oligos for gRNA oligo duplex	Sequences (5’ to 3’)
Insig1-gRNAs	CACCGGGGCGCGGGGTCAGCGTCCG
Insig1-gRNAa	AAACCGGACGCTGACCCCGCGCCCC
underline shows targeting sequence of 161–177 from translation initiation site in Insig1 on genome	
Insig2-gRNAups	CACCGGACAGTTGAGCTTTTCAGCT
Insig2-gRNAupa	AAACAGCTGAAAAGCTCAACTGTCC
underline shows targeting sequence of −72–−53 from translation initiation site in Insig2 on genome	
Insig2-gRNAdowns	CACCGGCATCTTTTCTTCTGCA
Insig2-gRNAdowna	AAACTGCAGAAGAAAAGATGCC
underline shows targeting sequence of 194–210 from translation initiation site in Insig2 on genome	

Table S3 Sequences of primers for qPCR

Targets	Forward primers (5’ to 3’)	Reverse primers (5’ to 3’)
**Chinese hamster**		
18s	TTCCGATAACGAACGAGACTCT	TGGCTGAACGCCACTTGTC
Chop	GGGAGCTGGAAGCCTGGTAT	GGGACCCCCATTTTCATCTG
Chac1	CTGTGGATTTTTGGGTACGG	CCCCTATGGAAGGTGTCTCC
Trb3	ATGCGAGCCACCCCTCTGGT	GGCCCAGGTTCGGGTCCTCT
Asns	TACAACCACAAGGCGCTACA	AAGGGCCTGACTCCATAGGT
Psat1	GGTGGTGATCGTCCGTGATG	GTTCCCAGCCTGCACTTTGT
Gadd34	CCTGGTCTGCAAAGTGCTGAT	CCAGCTCAGTCACTCCCTCTTC
Hri (Eif2ak1)	AGAGGTTCGGTACCACCTGATG	CCACAGGGAGAGCTCACACA
Pkr (Eif2ak2)	GCCTCTCCAGCAGCTTGTCT	CACTTTGCGGTGCTGACAGA
Perk (Eif2ak3)	TCCCTCTGCCGACGATCA	CAAGACAGGAGTCCTAGAAGGAGAA
Gcn2 (Eif2ak4)	AAACCCCGGGCGAACTC	CTTCGCCACTACAGACAGAATACTG
Abca1	GCTCTGGTGTTCAGCCTAAT	CTGGTTAGAGCATTCAGGAGTT
Abcg1	GGGATCAGAACAGTCGCCTG	CGAGGTCTCTCTTATAGTCAGCGTC
**Human**		
18S	ACCGCAGCTAGGAATAATGGA	GCCTCAGTTCCGAAAACCA
INSIG1	CCTTTGGTGGACATTTGATCGT	GCGTAGCTAGAAAAGCTATGGTGAT
INSIG2	TAATGCGGTGTGTAGCAGTCT	GTCCAATGGATAGTGCAGCCA-
CHOP	AGGGAGAACCAGGAAACGGAAACA	TCCTGCTTGAGCCGTTCATTCTCT
TRB3	TCAAGCTGTGTCGCTTTGTC	GAATCATCTGGCCCAGTCAGC

Table S4 Sequences of siRNAs

Target	Sence (5’ to 3’)	Antisence (5’ to 3’)
**Chinese hamster**		
Hri (*Eif2ak1*)	GCAGCUCCUCCAUUAUAUUTT	AAUAUAAUGGAGGAGCUGCTT
Pkr (*Eif2ak2*)	CCAUGGACUCCAGGUUUAATT	UUAAACCUGGAGUCCAUGGTT
Perk (*Eif2ak3*)	GGUAUAUCUGUUCUGCCUUTT	AAGGCAGAACAGAUAUACCTT
Gcn2 (*Eif2ak4*)	GGGAAUGAUUCAUCGGGAUTT	AUCCCGAUGAAUCAUUCCCTT

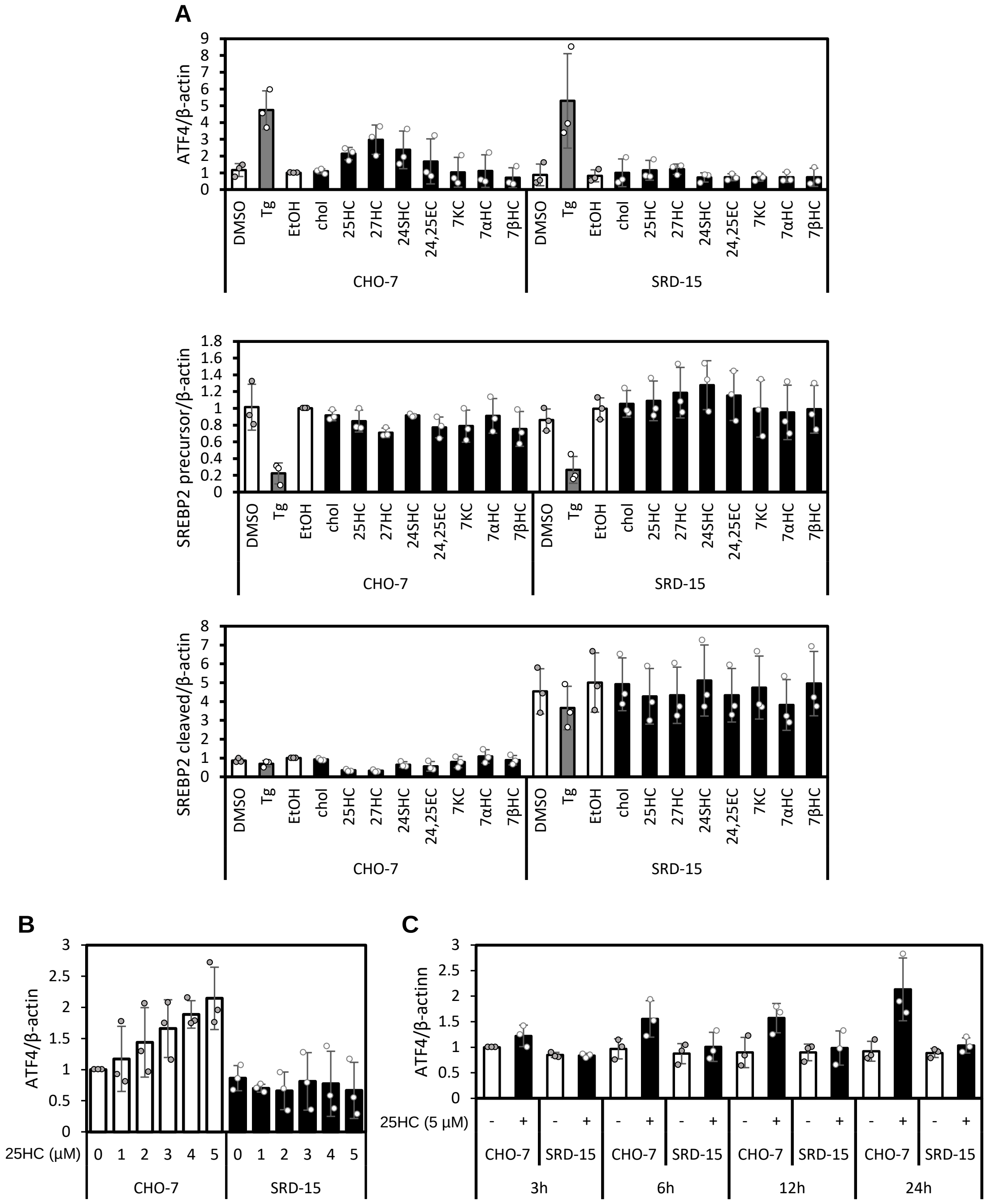

Fig. S1. Quantification of protein expression levels in Fig. 1. Signal intensities in Fig.1B–D (A–C, respectively) were quantified by densitometric scanning. Signal intensities of each protein were normalized first to β-actin in each sample expressed relative to expression in CHO-7 cells after 24 h incubation with EtOH and 0 μM 25HC (A and B) or CHO-7 cells after 3 h incubation without 25HC treatment (C). The data shows mean ± SD obtained in the biological triplicate assay with the individual data points were indicated in a circle.

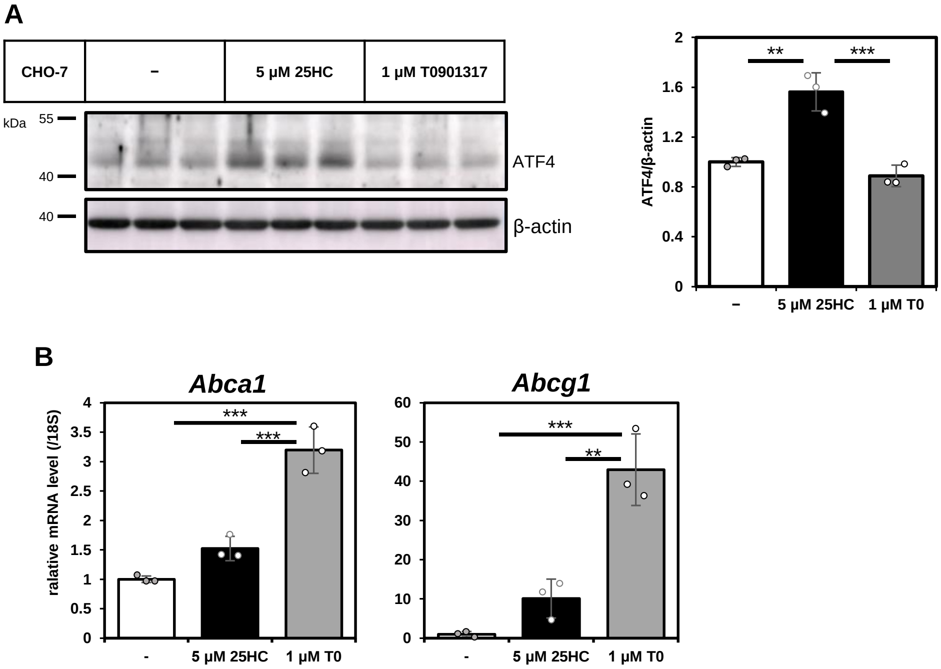

Fig. S2. 25HC induced ATF4 independently of LXR activation. CHO-7 cells were treated with 5 μM 25HC or 1 μM T0901317 (T0) for 24 h. (A) *Left panel*, whole-cell lysates were subjected to SDS-PAGE, followed by immunoblot analysis with specific antibodies as indicated. *Right panel*, Quantification of ATF4 relative to β-actin. Signal intensity was quantified by densitometric scanning. The signal intensity of ATF4 was normalized first to β-actin in each sample and expressed relative to expression in the negative control (−). (B) The mRNA of LXR target genes were measured using appropriate primers by RT-qPCR. The data shows mean ± SD obtained in the biological triplicate assay with the individual data points were indicated in a circle. ***P* < 0.01 and ****P* < 0.001.

A

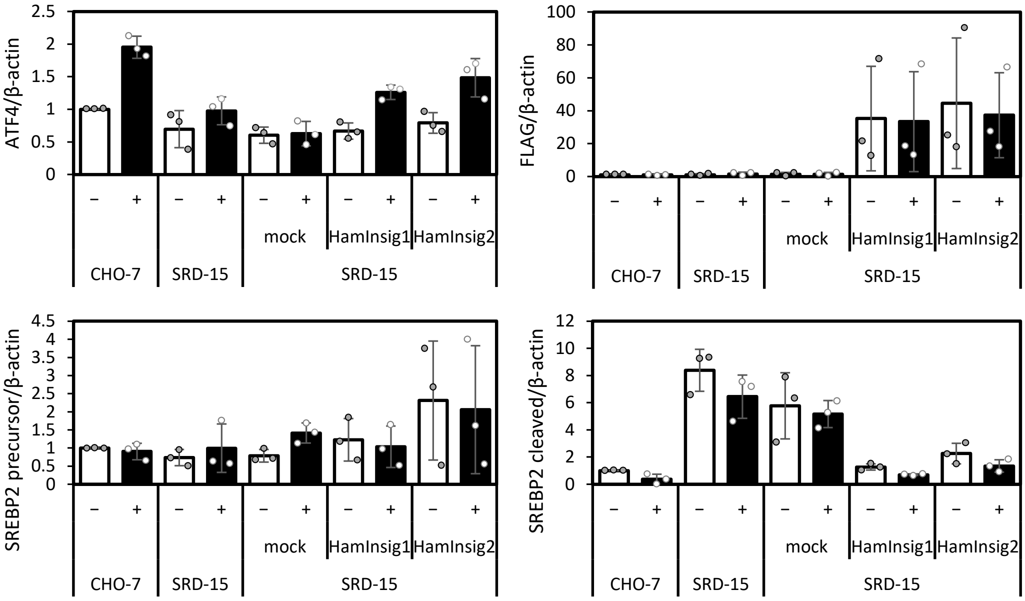
ATF4/β-actin
FLAG/β-actin
SREBP2 precursor/β-actin
SREBP2 cleaved/β-actin
− + − + − + − + − +
mock
HamInsig1
HamInsig2
CHO-7
SRD-15
SRD-15

B

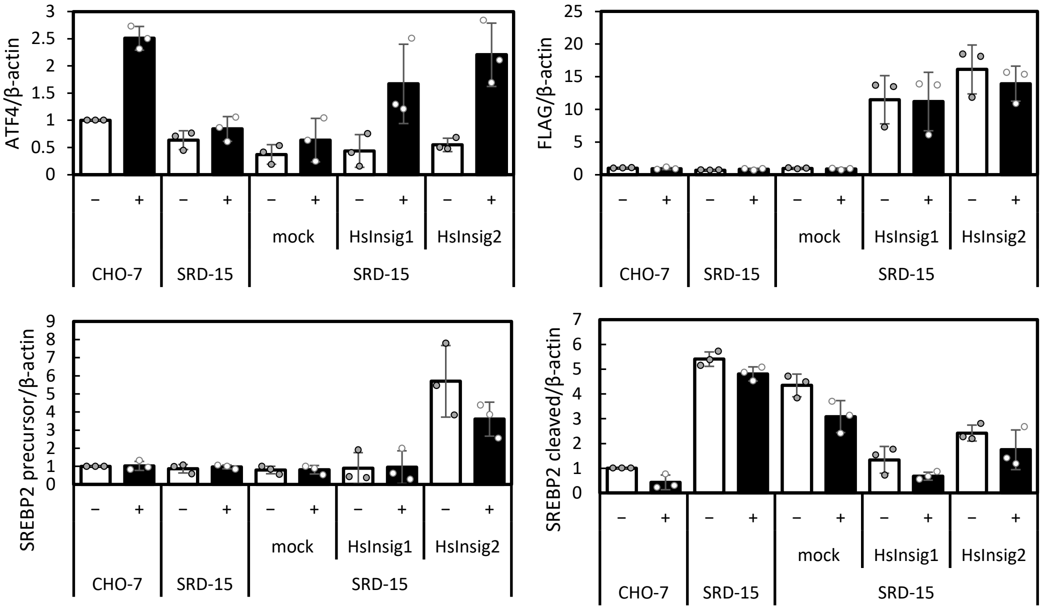
ATF4/β-actin
FLAG/β-actin
SREBP2 precursor/β-actin
SREBP2 cleaved/β-actin
− + − + − + − + − +
mock
HsInsig1
HsInsig2
CHO-7
SRD-15
SRD-15

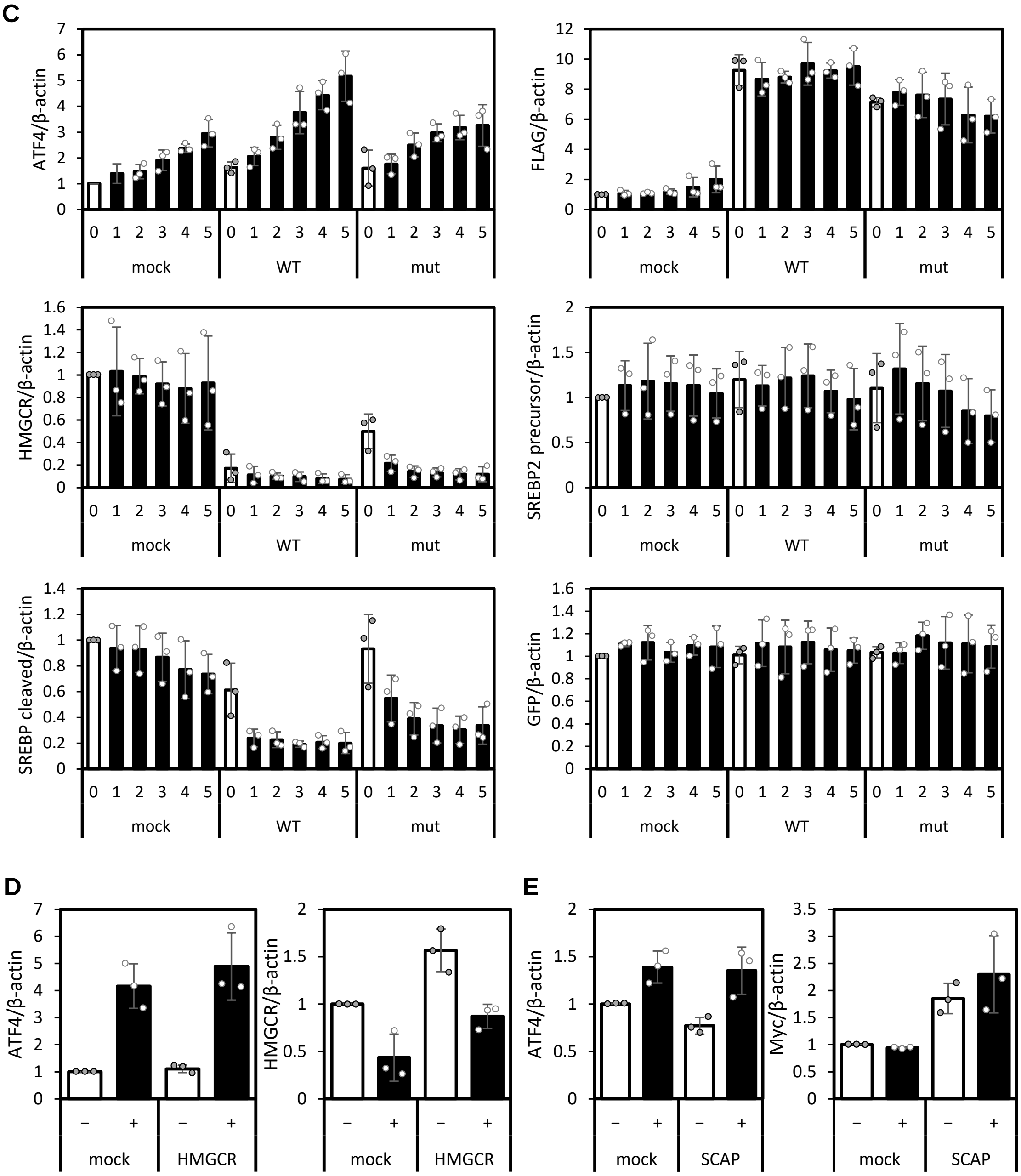

Fig. S3. Quantification of protein expression levels in Fig. 2. Signal intensities in Fig.2A–D (A–D, respectively) were quantified by densitometric scanning. Signal intensities of each protein were normalized first to β-actin in each sample expressed relative to expression in CHO-7 cells after 24 h incubation without 25HC (A and B) or CHO-7 cells transfected with mock vector after 24 h incubation without 25HC treatment (C–E). The data shows mean ± SD obtained in the biological triplicate assay with the individual data points were indicated in a circle.

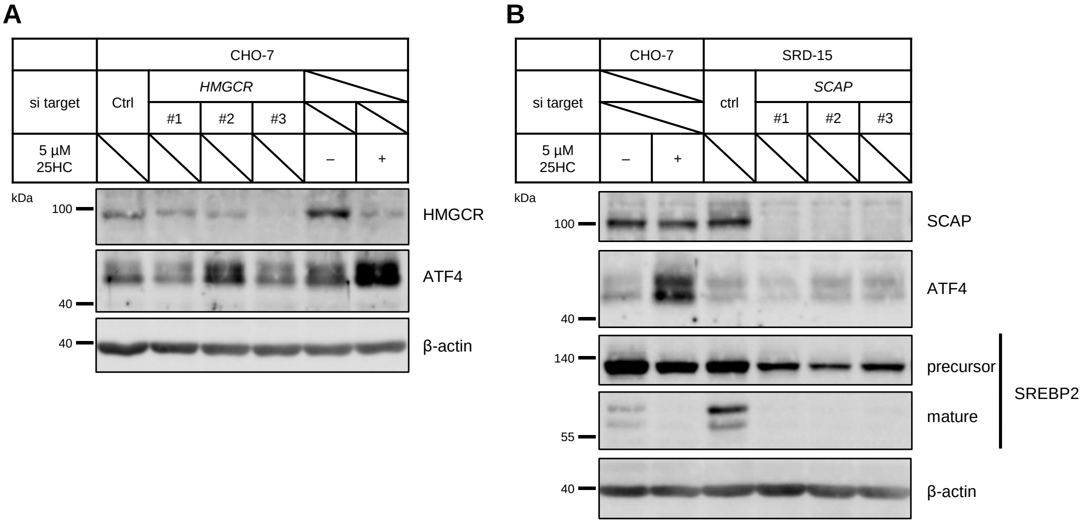

C

Target	Sence (5' to 3')	Antience (5' to 3')
Hmgcr		
ctrl	GAGCUUGCCAAUUGUCGGACGGUUA	UAACCGUCCGACAAUUGGCAAGCUC
#1	GGGACGGUGACACUUACCAUCUGUA	UACAGAUGGUAAGUGUCACCGUCCC
#2	GAGGGUCGUCCAAUUUGGCAGCUUA	UAAGCUGCCAAAUUGGACGACCCUC
#3	UGCCCGAAUUGUGUGUGGUACUGUA	UACAGUACCACACACAAUUCGGGCA
Scap		
ctrl	GAGAUAGCUCUCGGACUUCUAAACA	UGUUUAGAAGUCCGAGAGCUAUCUC
#1	GAGAAGAUAUCUCAGGCCUUCUACA	UGUAGAAGGCCUGAGAUAUCUUCUC
#2	GGAACAGGACCUGUGGAAUUCUCUA	UAGAGAAUUCCACAGGUCCUGUUCC
#3	CACAGGCAUCAAGCUUUACUCCAUU	AAUGGAGUAAAGCUUGAUGCCUGUG

Fig. S4. 25HC induced ATF4 independently of HMGCR and SCAP expression. (A, B) CHO-7 or SRD-15 cells were transfected with control and Hmgcr- (A) or Scap- (B) targeting siRNA for 16 h, respectively. The transfected cells were further incubated for 48 h. CHO-7 cells without transfection of siRNAs were treated with 5 µM 25HC for 24 h. Whole-cell lysates were subjected to SDS-PAGE, followed by immunoblot analysis with specific antibodies as indicated. (C) The list of Stealth RNAi™ siRNAs (Thermo Fisher Scientific) targeting Chinese hamster *Hmgc* (XM_007648619), *Scap* (NM_001244036), and their negative control siRNAs.

```
INSIG1          MPRLHDHFWSCSCAHSARRRGPPRASAAGLAAKVGEMINVSVSGPSLLAAHGAPDADPAP 60
INSIG2          ------------------------------------------------MAEGETES-PGP 11
                                                                 * *     * *

INSIG1          RGRSAAMSGPEPGSPYPNTWHHRLLQRSLVLFSVGVVLALVLNLLQIQRNVTLFPEEVIA 120
INSIG2          KKCGPYISSVTSQSVN-------LMIRGVVLFFIGVFLALVLNLLQIQRNVTLFPPDVIA 64
                       *     *         *  *  ***  ** ******************  ***

INSIG1          TIFSSAWWVPPCCGTAAAVVGLLYPCIDSHLGEPHKFKREWASVMRCIAVFVGINHASAK 180
INSIG2          SIFSSAWWVPPCCGTASAVIGLLYPCIDRHLGEPHKFKREWSSVMRCVAVFVGINHASAK 124
                 *************** ** ******** ************ ***** ************

INSIG1          LDFANNVQLSLTLAALSLGLWWTFDRSRSGLGLGITIAFLATLITQFLVYNGVYQYTSPD 240
INSIG2          VDFDNNIQLSLTLAALSIGLWWTFDRSRSGFGLGVGIAFLATVVTQLLVYNGVYQYTSPD 184
                 ** ** ********** ************ ***  ******  ** *************

INSIG1          FLYIRSWLPCIFFSGGVTVGNIGRQLAMG----VPEKPHSD 277
INSIG2          FLYVRSWLPCIFFAGGITMGNIGRQLAMYECKVIAEKSHQE 225
                *** ********* ** * *********       ** *
```

Fig. S5. A sequence alignment of human INSIG1 and INSIG2 by CLUSTALW. Asterisk shows completely conserved amino acids. Red arrowhead indicates Phe115 and Thr136 in INSIG2 that are important residues for oxysterol binding. The sequences used are human INSIG1 (Accession no. O15503) and INSIG2 (Accession no. Q9Y5U4).
